# Filamentous *Phytophthora* Pathogens Deploy Effectors to Interfere With Bacterial Growth and Motility

**DOI:** 10.3389/fmicb.2020.581511

**Published:** 2020-09-30

**Authors:** Ji Wang, Danyu Shen, Chengcheng Ge, Yaxin Du, Long Lin, Jin Liu, Tian Bai, Maofeng Jing, Guoliang Qian, Daolong Dou

**Affiliations:** Key Laboratory of Plant Immunity, Department of Plant Pathology, College of Plant Protection, Nanjing Agricultural University, Nanjing, China

**Keywords:** *Phytophthora*, effector, bacterial growth, motility, contact-dependent growth inhibition

## Abstract

*Phytophthora* comprises a group of filamentous plant pathogens that cause serious crop diseases worldwide. It is widely known that a complex effector repertoire was secreted by *Phytophthora* pathogens to manipulate plant immunity and determine resistance and susceptibility. It is also recognized that *Phytophthora* pathogens may inhabit natural niches within complex environmental microbes, including bacteria. However, how *Phytophthora* pathogens interact with their cohabited microbes remains poorly understood. Here, we present such an intriguing case by using *Phytophthora*–bacteria interaction as a working system. We found that under co-culture laboratory conditions, several *Phytophthora* pathogens appeared to block the contact of an ecologically relevant bacterium, including *Pseudomonas fluorescence* and a model bacterium, *Escherichia coli*. We further observed that *Phytophthora sojae* utilizes a conserved Crinkler (CRN) effector protein, PsCRN63, to impair bacterial growth. *Phytophthora capsici* deploys another CRN effector, PcCRN173, to interfere with bacterial flagellum- and/or type IV pilus-mediated motility whereas a *P. capsici*-derived RxLR effector, PcAvh540, inhibits bacterial swimming motility, but not twitching motility and biofilm formation, suggesting functional diversification of effector-mediated *Phytophthora*–bacteria interactions. Thus, our studies provide a first case showing that the filamentous *Phytophthora* pathogens could deploy effectors to interfere with bacterial growth and motility, revealing an unprecedented effector-mediated inter-kingdom interaction between *Phytophthora* pathogens and bacterial species and thereby uncovering ecological significance of effector proteins in filamentous plant pathogens besides their canonical roles involving pathogen–plant interaction.

## Introduction

*Phytophthora* spp., a genus of soilborne phytopathogenic oomycetes, infects a wide range of plants and crops to threaten crop production and cause devastating damage to the ecosystem globally (Kamoun et al., 2015). Several species of *Phytophthora*, including *Phytophthora sojae*, *Phytophthora capsici*, and *Phytophthora infestans*, have been described as causal agents of soybean root and stem rot, pepper and cucumber blight, and potato and tomato late blight, respectively (Tyler, 2007; Haas et al., 2009; Lamour et al., 2012). Recent studies revealed that *Phytophthora* pathogens utilize effector proteins to manipulate plant immunity and promote infection (Dou and Zhou, 2012; Lamour et al., 2012; He et al., 2020). Among various types of effectors, RxLR and Crinkler (CRN) represent two major groups of cytoplasmic effectors that have been well-characterized (Tyler, 2007; Haas et al., 2009; Petre and Kamoun, 2014). For instance, the *P. infestans* RxLR effector Pi06280 interacts with host susceptibility factor NRL1 to enhance the association between NRL1 and SWAP70, a guanine nucleotide exchange factor, by which Pi06280 promotes the proteasome-mediated degradation of SWAP70, leading to the suppression of plant immunity (He et al., 2018). In *P. capsici*, an RxLR effector, Avh103, targets host EDS1 and suppresses plant immunity, probably through promoting the disassociation of the EDS1–PAD4 complex (Li et al., 2020). In *P. sojae*, two close CRN homologous effectors, PsCRN63 and PsCRN115, could interact with plant catalases to inversely trigger H_2_O_2_-mediated defense responses in plants (Liu et al., 2011; Zhang et al., 2015). It is noteworthy that despite *Phytophthora* pathogens containing a complex effector repertoire, only few members have certain functions as plant immunity regulators (Amaro et al., 2017; Wang and Jiao, 2019; He et al., 2020). The functionality of most effector proteins in *Phytophthora* still remains to be uncovered.

In nature, it is believed that the filamentous *Phytophthora* pathogens cohabited with other microbes, such as bacteria (Berendsen et al., 2012). However, how *Phytophthora* pathogens communicate or interact with their niche’s related microbes to gain ecological adaptation remains unknown. In the bacterial kingdom, it is well-known that a wide range of Gram-negative bacteria could deploy type VI secretion system (T6SS) or type IV secretion system (T4SS) to inject toxic effector proteins into neighbor cells of bacterial or eukaryotic preys to interfere with or even kill them, leading to a contact-dependent growth inhibition (CDI) or inter-kingdom competition (Ma et al., 2014; Mariano et al., 2018; Sgro et al., 2019). These previous studies collectively lead us to hypothesize that the filamentous *Phytophthora* pathogens may also employ an effector-mediated approach to achieve its previously unidentified patter of communications/interactions with their niche’s related microbes.

In the present study, we observed that several *Phytophthora* plant pathogens appeared to block the contact of two bacterial species in a developed co-culture system, revealing that an “unfriend” interaction pattern occurs between *Phytophthora* and particular bacterial species. We further found that *P. sojae*, a causal pathogen of soybean root and stem rot, could utilize a conserved CRN effector protein to interfere with bacterial growth, which acts as a mechanism explaining how *Phytophthora* pathogens block the contact of bacterial cells. We also found that *Phytophthora* pathogens secreted two unrelated effectors, PcCRN173 and PcAvh540, to interfere with bacterial motility. We hypothesized that inhibition of bacterial motility may represent another mechanism employed by *Phytophthora* pathogens to avoid the contact of bacterial cells. Thus, our studies provide a first case showing that the filamentous *Phytophthora* pathogens could deploy the effectors to manipulate bacterial growth and motility, revealing an unrecorded effector-mediated inter-kingdom interaction between filamentous pathogens and bacteria.

## Materials and Methods

### Microbial Strains and Plant and Growth Conditions

*P. sojae* (P6497), *P. capsici* (LT263), and *Phytophthora nicotianae* (Pp025) strains were routinely cultured on 10% vegetable (V8) juice medium at 25°C in the dark. A *Botrytis cinerea* strain was cultured on PDA medium at 25°C in the dark. *Escherichia coli* DH5α, BL21(DE3), and MG1655 strains were cultured in LB medium at 37°C. *Pseudomonas fluorescence* 2P24, *Agrobacterium tumefaciens* GV3101, and *Lysobacter enzymogenes* OH11 strains were cultured in LB medium at 28°C. *Nicotiana benthamiana* plants were grown for 4–6 weeks at 25°C under a 16 h light/8 h dark photoperiod in a greenhouse.

### Plasmid Construction

Constructs and primers used for plasmid construction in this study are documented in additional file Supplementary Data Sheets 1, 2. AvrRxo1 was cloned using the DNA from *Xanthomonas oryzae* pv. *oryzae* RS105, PsCRN63, and PsCRN115 were cloned from the cDNA of *P. sojae* strain P6497, PnSCP47 was cloned using the cDNA of *P. nicotianae* Pp025, and other effectors were PCR amplified from *P. capsici* strain LT263. For arabinose-induced gene expression, corresponding PCR products were inserted into pBAD/Myc-HisA plasmid. To generate constructs for RFP-labeled gene transient expression in *N. benthamiana*, the coding region (including signal peptide) of PsCRN63 and PsCRN115 was inserted into the pSuper-RFP vector, respectively. For transiently expressed effector’s protein extraction from *N. benthamiana*, the coding region (without signal peptide) of PsCRN63 and PsCRN115 was separately cloned into the pBinGFP2 vector. To make constructs for twitching motility assay, the coding region of PcCRN173 and PcAvh540 was PCR-amplified to the PBBR1-MCS5 vector. All generated plasmids were validated by sequencing by GenScript, Inc. (Shanghai, China).

### *Phytophthora* and LacZ-Labeled Bacterial Co-culture Assays

*P. sojae* (P6497), *P. capsici* (LT263), *P. nicotianae* (Pp025), and *B. cinerea* were cultured on the indicated solid medium first. After hyphae grew well, agar disks covered with mycelium were obtained with a hole punch. The same numbers of agar disks for each *Phytophthora* and *B. cinerea* were placed in the conical flask filled with filtered 20 ml 10% liquid V8 medium, shaken and cultured at 25°C in the dark. Mycelial pellets formed in 2–3 days. LacZ-labeled *P. fluorescence* 2P24 and *E. coli* DH5α bacteria were cultured for 12 h in LB medium. Bacterial cells were harvested by centrifugation and resuspended in sterilized ddH_2_O, and the optical densities of cell suspensions were adjusted to an OD_600_ of 0.5. Two hundred microliters of bacterial suspensions and 100 μl of 20 mg/ml X-gal were added to each conical flask with mycelial pellets. Then, *Phytophthora* and bacteria were co-cultured at 25°C in the dark with shaking for 3 days.

### Detection of Effector-Induced Toxicity in *E. coli* BL21

The arabinose inducible plasmid pBAD/Myc-HisA was introduced for toxic effector detection. *E. coli* strain BL21 (DE3) transformed with empty pBAD and pBAD-AvrRxo1 was cultured in LB broth for approximately 4 h. Exponentially growing cells were adjusted to an OD_600_ of 0.5 and serially diluted 10-fold. A 5 μl volume of each bacterial dilution was then spotted on solid LB agar supplemented with and without appropriate concentrations of L-arabinose. Plates were placed at 37°C overnight and photographed.

### Growth Curve Assays

Freshly cultured *E. coli* BL21 (DE3) strains carrying pBAD-based vectors were suspended to an OD_600_ of 0.03 and distributed to six replicate tubes: three replicate tubes for uninduced groups and three other tubes for arabinose-induced groups. Bacteria were induced with 2% L-arabinose at 0 h and incubated at 37°C with shaking. Cell growth was recorded every hour at OD_600_.

### Western Blot Assays for PsCRN63 and PsCRN115 Induction

*E. coli* BL21(DE3) strains transformed with pBAD-PsCRN63, pBAD-PsCRN115, or pBAD empty vector were grown in LB broth at 37°C for several hours until the optical density (OD_600_) reached 0.3. Final concentrations of 0.1% and 2% L-arabinose were used to induce gene expression. After 5 h of induction, bacterial cells were collected and lysed for western blot. Samples were separated by standard SDS-PAGE gels and transferred to a PVDF membrane and then blocked with a PBST solution containing 5% non-fat milk for 30 min in room temperature with 60 rpm shaking. Anti-His monoclonal antibody (Sigma-Aldrich) and anti-β-RNAP (β-RNA polymerase) antibody (Abcam) were separately added to the PBST solution and incubated at 4°C overnight. Then the membranes were washed three times with PBST, followed by incubation with goat anti-mouse IRDye 800CW antibody (Odyssey) and goat anti-rabbit IRDye 800CW antibody (Odyssey) in PBST solution with 5% non-fat milk at room temperature for 45 min. The membranes were washed three times with PBST and visualized using a LI-COR Odyssey scanner with excitation at 700 and 800 nm.

### Transient Expression Assays

*Agrobacterium*-mediated transient expression in *N. benthamiana* was performed. *A. tumefaciens* strain GV3101 harboring the indicated constructs was cultured in LB broth at 28°C for 24 h. Bacterial cells were harvested by centrifugation at 4,000 rpm for 10 min, washed three times in 10 mM MgCl_2_, resuspended in infiltration buffer (10 mM MES, 10 mM MgCl_2_, 150 μM acetosyringone, pH = 5.6) to an optical density (OD_600_) of 0.5, and incubated at 28°C in the dark for over 3 h. Five-week-old *N. benthamiana* were used for infiltration. For protein extraction experiment, leaf samples were harvested 36 h after infiltration. For effector protein and bacterial co-incubation assay, leaves were infiltrated with bacterial suspension at 3 days after infiltration.

### Confocal Laser Scanning

Small patches of *N. benthamiana* leaf were mounted in distilled water after 48 h of infiltration. Fluorescence was visualized using a Zeiss LSM 710 confocal microscope. The red fluorescence of pSuper-RFP constructs was excited at 561 nm. Leaves with well-expressed constructs were used for further GFP-tagged bacterial infiltration. Freshly cultured GFP-tagged *E. coli* bacteria were washed three times in distilled water and adjusted to an OD_600_ of 0.2 and then uniformly infiltrated in leaves expressing the indicated pSuper-RFP constructs. Twenty-four hours after infiltration, the bacterial survival condition was visualized. The GFP fluorescence was excited at 488 nm. Images were processed using a Zeiss LSM 710 confocal laser scanning microscope with a ×20 objective lens.

### Transient Expressed Effector Protein and Bacterial Co-culture Assays

Effector proteins were transiently expressed in *N. benthamiana* for 36 h; then total protein was extracted using protein extraction buffer (50 mM HEPES, 150 mM KCl, 1 mM EDTA, and 0.1% Triton X-100; pH 7.5) supplemented with a 1 mM protease inhibitor cocktail (Roche). Protein was filtrated by bacteria filters and then mixed with bacterial suspension (OD_600_, 0.2) in the same volume. The protein and bacterial mixtures were incubated at 28°C with shaking. Samples were taken at 0, 1, 2, 3, 4, and 6 h of incubation. For each sample, five dilution series were prepared from the same original suspension, and 10 μl of the suspension was spotted on LB plates. Each sample was repeated three times. Plates were placed at 28°C overnight.

### Motility Assays

The test of MG1655 swimming motility was performed as described earlier (Ryjenkov et al., 2006). *E. coli* strains were grown in LB broth overnight, and 2 ml of cultures was spotted onto soft LB plates which contain 0.25% agar. Expression of the pBAD/Myc-HisA vector was induced with 0.1% L-arabinose. Swimming motility was observed after 6 h of incubation at 37°C. The twitching motility assay of OH11 was performed according to our earlier study (Zhou et al., 2015). In brief, *L*. *enzymogenes* strains carrying the pBBR1-MCS5 vector (Kovach et al., 1995) were inoculated at the edge of a sterilized coverslip containing 1/20 tryptic soy agar (TSA) with 1.8% agar. The margin of bacterial colonies on the microscope slide was observed after 24 h of incubation at 28°C. The twitching motility was specified by the observation of motile cells or cell clusters growing away from the primary colony.

### Biofilm Formation Assays

Biofilm formation assay in *L. enzymogenes* was performed as in our earlier study (Xia et al., 2018). Firstly, *L. enzymogenes* strains were grown in LB broth at 28°C with shaking at 200 rpm. When the OD_600_ reached 1.0, 80 μl bacterial culture of each strain was transferred into 12-well plates containing 4 ml LB broth in each well. The 12-well plates were grown at 28°C for 72 h without shaking. Then the cultures were removed, and the respective well was washed three times by using sterilized water, followed by the addition of 5 ml crystal violet (CV). After 15 min, the CV was removed, and each well was washed by using sterilized water three times. Finally, the 12-well plates were put at 37°C for drying, and CV-stained biofilms were dissolved in 4 ml elution buffer (50% H_2_O, 40% methanol, 10% glacial acetic acid). The biofilm formation was quantified by measuring OD_575_ with a spectrometer. Three replicates for each sample were used in this assay.

## Results

### Several *Phytophthora* Pathogens Appear to Block Contact of Bacterial Cells in a Co-culture System

**FIGURE 1:**
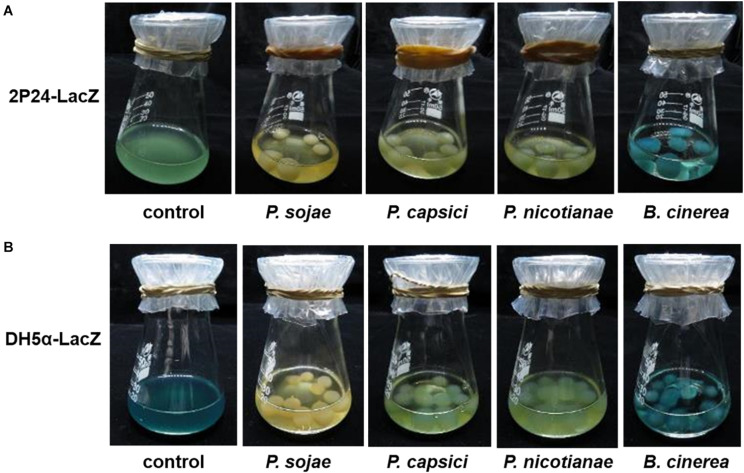
The selected *Phytophthora* pathogens appeared to block the contact of the LacZ-labeled *P. fluorescence* **(A)** and *E. coli* **(B)** under co-culture conditions. The LacZ-labeled bacterial strains could become blue in the presence of X-gal. *P. sojae*, *P. capsici*, *P. nicotianae*, and *B. cinerea* were first cultured in solid medium and then transferred to liquid medium, shaken and cultured at 25°C in the dark. As mycelial pellets formed, LacZ-labeled bacterial suspensions were added to the medium for co-culture in the presence of X-gal solution. Under the co-culture conditions, if the bacterial cells contacted with the pathogen hypha, the hypha will show a blue appearance due to attachment of “blue” bacterial cells. “Control” means presence of only bacterial cells. *P. sojae*, *P. capsici*, *P. nicotianae*, and *B. cinerea* correspond to *Phytophthora sojae*, *Phytophthora capsici*, *Phytophthora nicotianae*, and *Botrytis cinerea*, respectively.

To mimic a natural interaction between plant *Phytophthora* pathogens and ecologically relevant bacteria in the laboratory, we selected three representative plant *Phytophthora* pathogens (*P. sojae*, *P. capsici*, and *P. nicotianae*) to perform a co-culture assay with a niche-associated widespread bacterial species, *P. fluorescence*. To facilitate our observation, we generated a LacZ-labeled *P. fluorescence* strain (2P24-LacZ) that could turn blue in the presence of X-gal (5-bromo-4-chloro-3-indolyl β-D-galactopyranoside). With the co-culture of this LacZ-labeled strain with the *Phytophthora* pathogens described above, it is interesting to observe that all the selected *Phytophthora* pathogens blocked the attachment of 2P24-LacZ, as no-blue or slightly blue bacterial cells could be detected around the hypha, while another filamentous fungal pathogen, *B. cinerea*, was observed to be attached well by the same bacterium (Figure 1A). This phenomenon was further validated by a model bacterium, a LacZ-labeled *E. coli* strain DH5α (pMD19-T) (Figure 1B). These results suggest that the filamentous *Phytophthora* pathogens seem to deploy previously uncharacterized factors to block the contact of bacterial cells. Observation of such an intriguing inter-kingdom interaction motivates us to uncover the underlying factors and/or mechanisms utilized by *Phytophthora* pathogens.

### PsCRN63 Is a *Phytophthora* CRN Effector Toxic to Bacterial Cells

**FIGURE 2:**
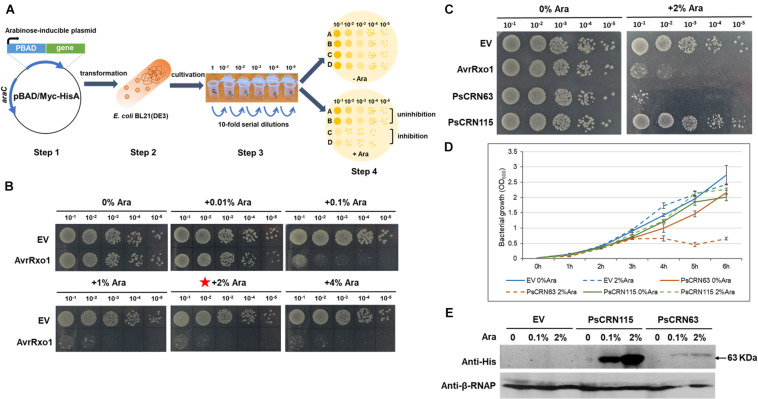
Induced expression of PsCRN63 caused toxicity to *E. coli* cells based on a generated genetic screen system. **(A)** A schematic illustration of the genetic screen system evaluating the toxicity of a given effector gene in *E. coli* cells. The effector gene was cloned into the pBAD/Myc-HisA vector (step 1), followed by transformation into the *E. coli* BL21 cells (step 2), in which the expression of the effector gene was driven by an arabinose-inducible promoter. After cultivation and serial dilutions (step 3), these transformed bacterial cells were spotted on the LB agar plates with (+Ara) and without (−Ara) arabinose (step 4) to observe their growth, which serves as an indication to evaluate the toxicity of the given effector gene. **(B)** Validation of the genetic screen system by using a *X. oryzae* T3SS (type III secretion system) effector protein, AvrRxo1 with known antibacterial activities. The toxicity to *E. coli* cells induced by the *AvrRxo1* gene was arabinose dose dependent compared to the vector control (EV). The red star indicates that this concentration was used in the following assay. **(C)** Induced expression of the *PsCRN63* gene but not its close homologous gene, *PsCRN115*, caused toxicity to *E. coli* cells. **(D)** Evaluation of the growth curve of *E. coli* cells carrying the given effector genes. *E. coli* BL21(DE3) cultures (*OD*_600_ = 0.03) transformed with pBAD-*PsCRN63*, pBAD-*PsCRN115*, or empty pBAD vector were induced with 2% L-arabinose at 0 h. Cell growth was recorded every hour at OD_600_. Data are the means ± SEM of three independent experiments. Experiments were repeated at least three times on different days. **(E)** Western blotting showing the detectable expression of the PsCRN63 and PsCRN115 genes in the *E. coli* cells after induction by arabinose. Anti-His antibodies were used to detect PsCRN63 and PsCRN115; the RNA polymerase β-subunit served as a loading control.

As no-blue/slightly blue bacterial cells were observed in the co-cultured *Phytophthora* pathogen hypha, we thus hypothesized that *Phytophthora* pathogens may have a capacity to interfere with bacterial growth by secreting factors such as the effector proteins. To rapidly test the toxicity of *Phytophthora* effectors to bacterial cells, we developed a model *E. coli*-based inducible system, in which the expression of each effector encoding gene was driven by an arabinose-inducible promoter (Figure 2A). To test whether this system could work properly, we first employed a bacterial effector gene, *AvrRxo1*, from *X. oryzae* as a control, whose product is known to be toxic to *E. coli* cells (Triplett et al., 2016). As expected, expression of *AvrRxo1* under the arabinose-inducible promoter caused toxicity to the test *E. coli* BL21 cells, and this toxic effect was shown to be arabinose dose dependent (Figure 2B). The concentration of 2% Ara was used for the following toxic effector screening. Using this generated system, we first tested whether the CRN effector, PsCRN63, from *P. sojae* is toxic to *E. coli* cells, because we previously already found that the recombinant PsCRN63–His fusion protein was hardly purified in *E. coli* BL21 (Zhang et al., 2015). Indeed, we found that inducible expression of the *PsCRN63* gene in *E. coli* BL21 cells caused toxicity and thus inhibited the bacterial growth (Figure 2C), which was further confirmed by the growth curve test in liquid medium (Figure 2D). Expression of *PsCRN63* in *E. coli* BL21 cells was also detected by western blot assays (Figure 2E). To explore whether this finding is biologically relevant, we further carried out a large-scale screen (40 effector proteins in total) by using the developed system, and we indeed found that PcCRN230 from *P. capsici* is another CRN effector toxic to *E. coli* (Supplementary Figures 1, 2). During the screen, we are surprised to find that inducible expression of PsCRN115, a homolog of PsCRN63, did not show a toxic effect to BL21 cells (Figure 2C). This unique observation motivated us to use “PsCRN63–PsCRN115” as a comparative pair to further confirm their toxicity/non-toxicity to bacterial cells.

**FIGURE 3:**
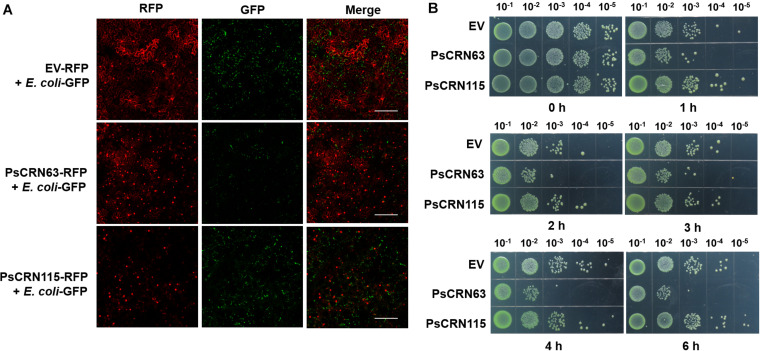
PsCRN63 directly inhibited bacterial growth. **(A)** Transient expression of the *PsCRN63*-RFP fusion gene in plant cells inhibited bacterial growth by using the GFP-labeled *E. coli* as a representative example. Tobacco leaves were infiltrated with *A. tumefaciens* strain GV3101 carrying *PsCRN63*-pSuper-RFP, *PsCRN115*-pSuper-RFP, or empty vector (*OD*_600_ = 0.5). The leaves were infiltrated with GFP-labeled *E. coli* (*OD*_600_ = 0.2) after 3 days of expression of the genes indicated. The confocal images were taken using a confocal laser scanning microscope (LSM 710 META, Zeiss, Germany) at 24 h, with excitation wavelengths of 488 nm (GFP) and 561 nm (RFP). Scale bars = 200 μm. The experiments had more than three biological repeats with similar results. Both the empty vector (EV-RFP) and the *PsCRN115*-RFP fusion gene were used as negative controls. **(B)** The plant-derived PsCRN63 proteins inhibited the growth of the test *E. coli* cells. pBinGFP2-*PsCRN63*, pBinGFP2-*PsCRN115*, and pBinGFP2 empty vector were transiently overexpressed in tobacco leaves for protein extraction. The transiently expressed crude proteins of PsCRN63 and PsCRN115 were mixed with bacterial suspension (OD_600_, 0.2) in the same volume. The protein and bacterial mixtures were incubated at 28°C with shaking. Samples were taken at 0, 1, 2, 3, 4, and 6 h of incubation. Serial dilutions of the mixtures were spotted on LB plates and cultured at 28°C overnight. Each sample was repeated three times.

Since the recombinant PsCRN63–His fusion proteins are difficult to purify with a desirable content in *E. coli*, we thus first adopted a plant-derived system, in which PsCRN63-RFP was transiently expressed in the leaves of *N. benthamiana*, followed by infiltrating the GFP-labeled *E. coli* cells to the same leaves to explore whether the plant-expressed PsCRN63 has an ability to inhibit bacterial growth. Using this approach, we again found that transient expression of PsCRN63 but not PsCRN115 in plants inhibited the growth of the GFP-labeled *E. coli* BL21 (Figure 3A), which is consistent with our earlier results shown in Figures 1, 2C. To further validate the above observations and exclude the possibility that suppression of bacteria is caused by PsCRN63-triggered plant immunity, we collected the plant-expressed proteins of PsCRN63 and PsCRN115 to perform a plate-based bacterial inhibition assay. Again, we observed that the plant-expressed PsCRN63, but not PsCRN115, directly inhibited the growth of *E. coli* in the culture (Figure 3B). These results collectively supported that PsCRN63 directly caused toxicity to bacterial cells and thus inhibited bacterial growth.

### The Residue Lysine of PsCRN63 at Position 329 Is Required for Its Induced Toxicity in *E. coli* Cells

**FIGURE 4:**
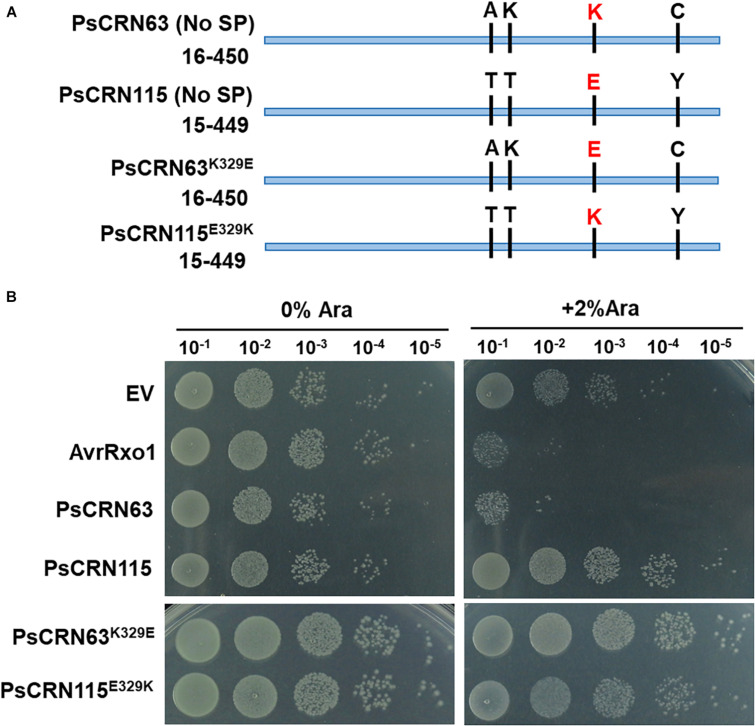
The residue lysine (K) of PsCRN63 at position 329 is required for its induced toxicity in *E. coli* cells. **(A)** A schematic illustration of four-amino difference between the sequence of PsCRN63 and its close homolog, PsCRN115. The residue lysine (K) at position 329 was highlighted in red and was randomly selected for site mutation. **(B)** Changing K329 to E329 of PsCRN63 (named PsCRN63^*K*329*E*^) abolished its induced toxicity to *E. coli* cells, while substitution of E329 to K329 of PsCRN115 (designated as PsCRN115^*E*329*K*^) did not confer its toxicity to *E. coli*. AvrRxo1 and empty vector were used as positive and negative controls, respectively. The same results were observed in at least three independent times.

Considering PsCRN115 only differs with PsCRN63 at four amino residues (Figure 4A), we thus tested whether these four amino residues could determine the toxicity of PsCRN63 to *E. coli*. Indeed, mutation of the residue lysine (Lys, K) at position 329 (named K329) of PsCRN63 to glutamic acid (Glu, E) that is possessed by PsCRN115 at the same position (named E329) abolished the induced toxicity to *E. coli*, while substitution of E329 of PsCRN115 by K329 did not confer an induced toxicity to *E. coli* (Figure 4B). These results suggest that while K329 plays key roles in maintaining the toxicity of PsCRN63 to bacterial cells, it is not the sole residue signature to distinguish the toxicity and non-toxicity between PsCRN63 and PsCRN115.

### One Homologous PsCRN63 Protein From *P. capsici* Caused Toxicity in *E. coli* Cells

**FIGURE 5:**
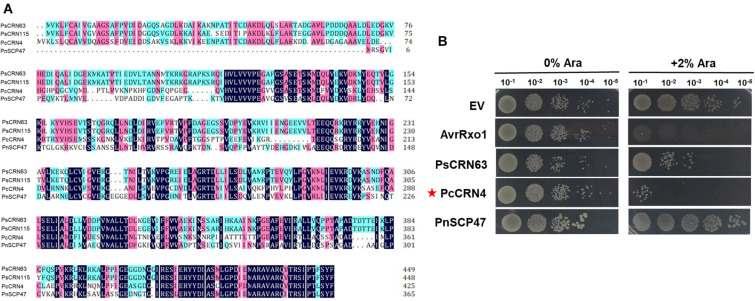
One homologous PsCRN63 protein from *P. capsici* caused toxicity to *E. coli* cells. **(A)** Sequence alignment of PsCRN63 and PsCRN115 with their selected homologs from *P. capsici* and *P. nicotianae*. PcCRN4 indicates *P. capsici* CRN4 (accession number: P0CV73.1), PnSCP47 represents *P. nicotianae* SCP47 (accession number: KUF94958.1). Identical amino acids are shaded in dark blue, amino acids with higher than 75% similarity are shaded in pink, and amino acids with higher than 50% similarity are shaded in light blue. **(B)** Induced expression of the *PcCRN4* gene (indicated by red stars) but not the *PnSCP47* caused toxicity to *E. coli* cells.

Our results above reveal that *P. sojae* could deploy PsCRN63 as a toxic effector against bacterial growth, which correlates with our earlier finding that the co-cultured *P. sojae* hypha blocked the contact of bacterial cells (Figure 1). To explore whether such an effector was conservatively distributed in and utilized by other plant *Phytophthora* pathogens tested in this study, we performed bioinformatic analyses and identified PcCRN4 from *P. capsici* and PnSCP47 from *P. nicotianae* as homologous proteins of PsCRN63 (Figure 5A). Subsequently, induced expression in *E. coli* BL21 identified that PcCRN4, but not PnSCP47, exhibited toxicity to bacterial cells (Figure 5B). These results reveal that PsCRN63 and its certain homologs from partial plant *Phytophthora* pathogens are indeed toxic to bacterial cells.

### PcCRN173 and PcAvh540 Interfere With Bacterial Motility

**FIGURE 6:**
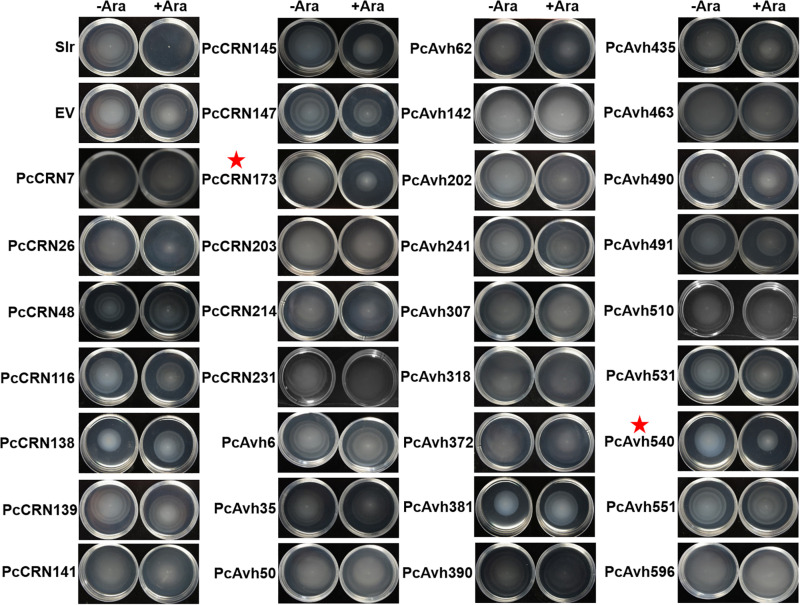
Large-scale screening revealed that induced expression of PsCRN173 and PsAvh540 indicated by red star in *E. coli* inhibited flagellum-mediated swimming motility. Expression of each given effector gene was driven by an arabinose-inducible promoter within the pBAD/Myc-HisA vector in *E. coli* MG1655. −Ara and +Ara indicate the absence and presence of 0.1% L-arabinose in the soft agar (0.35%) plates. Bacteria were incubated for 6 h at 37°C. The flagellum-mediated motility is indicated by the swimming halos formed in each plate. Slr, an abbreviation of Slr-1143 that is a well-characterized diguanylate cyclase inhibiting swimming motility, was used as a positive control. Three independent replicates were performed with similar results.

Besides toxicity, we are also interested to explore whether plant *Phytophthora* pathogens could utilize effector proteins to interfere with bacterial motility, thereby suppressing their movement/colonization and hence avoiding their contact. To test this hypothesis, each non-toxic effector (38 in total) was individually introduced into *E. coli* MG1655 that could display an easy-to-be-observed, flagellum-dependent swimming motility on semisolid agar plates. By using this system, we found that two *Phytophthora* effectors, PcCRN173 and PcAvh540, showed a remarkable role in inhibiting the swimming motility of MG1655, while 36 other effectors did not (Figure 6).

**FIGURE 7:**
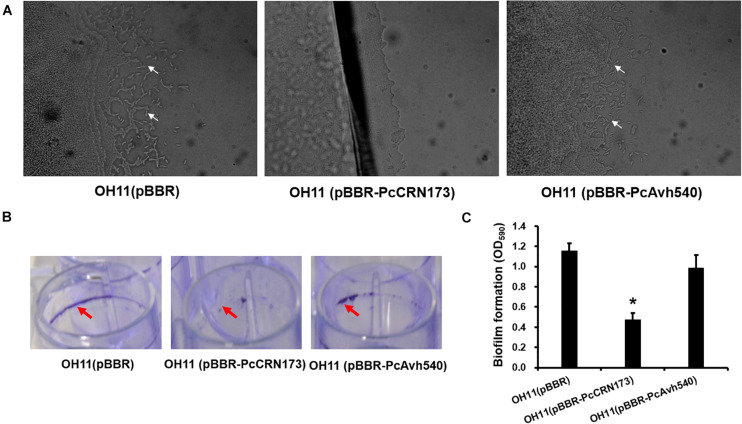
Differential involvements of PcCRN173 and PcAvh540 to twitching motility and biofilm formation in the non-flagellated *L. enzymogenes* OH11. **(A)** PcCRN173 but not PcAvh540 inhibited type IV pilus-mediated twitching motility. In 1/20 TSB agar medium, wild-type OH11 containing an empty vector exhibited twitching motility, as evidenced by the appearance of mobile cells (indicated by white arrows) at the colony margin, while OH11 containing PcCRN173 reveals no such capability. **(B)** PcCRN173 but not PcAvh540 inhibited biofilm formation. In LB liquid medium, wild-type OH11 containing an empty vector formed strong biofilms (indicated by red arrows) while OH11 containing PcCRN173 almost lost such abilities. **(C)** Quantification of biofilm formation formed by OH11 possessing an empty vector, PcCRN173, and PcAvh540. The absorbance value of OD_575_ was measured. Three replicates for each sample were tested. Asterisks (*) indicate significant differences (*P* < 0.05, Duncan’s test) relative to the empty vector control.

To further explore whether PcCRN173 and PcAvh540 also have a role in affecting flagellum-independent bacterial motile behavior, we cloned these two genes into a broad-host vector, pBBR1-MCS5, in which the expression of each gene was driven by a constitutive promoter. Subsequently, we introduced each construct into the *L. enzymogenes* OH11 (Qian et al., 2009) that is a non-flagellated strain but could exhibit type IV pilus (T4P)-driven twitching motility. The results of a twitching test showed that constitutive expression of PcCRN173 but not PcAvh540 completely inhibited the T4P-dependent twitching motility produced by the strain OH11 (Figure 7A). According to our earlier studies (Han et al., 2018), we have shown that twitching motility in strain OH11 correlates with the biofilm formation, a bacterial community state; we thus tested whether PcCRN173 could change the biofilm production in strain OH11. In agreement, we indeed found that the wild-type OH11 carrying PcCRN173 but not PcAvh540 significantly lowered the biofilm formation (Figures 7B,C).

## Discussion

In the past decades, the role and diverse mechanistic actions of effector proteins from filamentous pathogens in manipulating host immunity have been well illustrated (Oliva et al., 2010; Dou and Zhou, 2012; He et al., 2020). Unlike this traditional effector-mediated pathogen–host interaction, the present study discovered, for the first time, an effector-mediated inter-kingdom interaction mode between the filamentous *Phytophthora* pathogens and bacteria. We demonstrated that *Phytophthora* pathogens could likely block the contact of ecologically relevant bacterial species by secreting diverse effector proteins to interfere with bacterial growth and/or motility directly or indirectly (Figure 7), which may contribute to the ecology/infection of *Phytophthora* pathogens in natural niches. It is possible that using such a novel inter-kingdom interaction, the filamentous *Phytophthora* pathogens could alter environmental or host microbiota composition and/or dynamics to obtain niche nutrients in a maximum extent, which helps its free-living lifestyle or infection. However, it is known that *Phytophthora* pathogens commonly use haustoria to secrete a range of virulence effectors (Dou and Zhou, 2012; Wang et al., 2018), while it is unclear whether this structure also participates in the oomycete–bacteria interaction. Uncovering this point and other possible processes in depth will advance our knowledge on how effectors delivered by oomycete are involved in the interaction with cohabited bacteria in nature.

According to our results, we found that the plant-expressed PsCRN63 proteins exhibited a direct inhibitory effect on bacterial growth in medium plates, suggesting that this effector protein has a potential chance to directly target bacterial cell wall or enter into the cell’s periplasmic spaces or cytoplasm, where this effector achieves its toxicity by binding to an uncharacterized bacterial survival-required protein or compound. As a piece of supporting evidence, we already knew that bacteria could deliver toxic T6SS or T4SS effector proteins into the periplasmic space to degrade peptidoglycan or into the cytoplasm to degrade RNA of prey bacterial cells (Hayes et al., 2010; Hachani et al., 2016; Chassaing and Cascales, 2018), thereby inhibiting the growth of prey cells. In this aspect, the identified PsCRN63 could serve as an interesting probe for a detailed mechanistic study in the future to understand how the *Phytophthora* effectors archive antibacterial activity.

**FIGURE 8:**
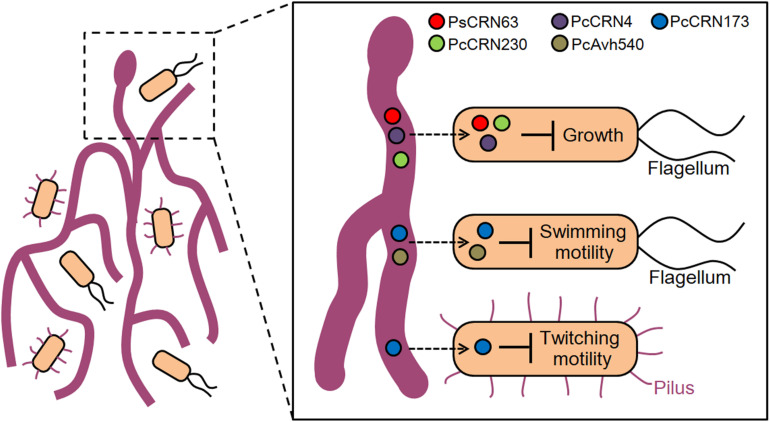
A proposed model illustrating an effector-mediated inter-kingdom interaction between *Phytophthora* pathogens and bacterial species. Based on the current results, it is proposed that *Phytophthora* pathogens seem to block the contact of bacterial species, representing an “unfriend” interaction pattern. To achieve such interactions, *Phytophthora* pathogens likely deploy several effector proteins in an uncharacterized entry/contact mechanism (indicated by a dashed arrow) to inhibit bacterial growth, flagellum-dependent swimming motility, and type IV pilus-mediated twitching motility, all of which likely help the filamentous pathogens to avoid the attachment of cohabited bacterial species and achieve better adaptation in environment.

Previous studies undoubtedly uncovered the important roles of flagella-dependent swimming motility and T4P-mediating twitching motility in bacterial chemotaxis, stress adaption, and virulence (Maier and Wong, 2015; Chang et al., 2016). Moreover, motility is also believed to play key roles in helping biocontrol bacteria (i.e., *P. fluorescence* 2P24) to move toward nearby microbial competitors or pathogens. For instance, we previously reported that the biological control agent *L. enzymogenes* OH11 utilizes T4P-driven twitching motility to move toward and establish a contact with filamentous pathogens and subsequently kill them by secreting antimicrobial secondary metabolites and abundant lytic enzymes (Han et al., 2015, 2018; Lin et al., 2020). With the above understandings along with our results in Figure 1, we hypothesized that the *Phytophthora* pathogens may deploy the CRN effectors, i.e., PcCRN173, to inhibit the twitching motility of OH11 and hence avoid the contact of this biocontrol bacterium, which likely represents a defense strategy of *Phytophthora* pathogens against bacterial infection. At the mechanistic level, we speculate that PcCRN173 may directly interact with a component or regulator of flagellum or pilus biogenesis, by which PcCRN173 could achieve its dual function in inhibiting both flagellum- and pilus-driven swimming and twitching motility (Figure 8).

In conclusion, the present study presents intriguing evidences to show that the plant *Phytophthora* pathogens could utilize effector proteins to interfere with bacterial growth and motility, revealing a crucial role of effector proteins in mediating the interaction of filamentous pathogens with its microbiome bacteria. Our results may open a gate to consider previously unrecognized ecological roles of filamentous pathogens’ effectors besides their well-characterized functions in pathogen–host interaction.

## Data Availability Statement

All datasets generated for this study are included in the article/Supplementary Material.

## Author Contributions

GQ and DD conceived the project and revised the manuscript. GQ, JW, and DD designed the experiments. JW, DS, CG, YD, LL, JL, and TB carried out the experiments. JW and DS analyzed the data and prepared the figures and tables. GQ, JW, and DS wrote the manuscript draft. All authors contributed to the article and approved the submitted version.

## Conflict of Interest

The authors declare that the research was conducted in the absence of any commercial or financial relationships that could be construed as a potential conflict of interest.

## Supplementary Material

Supplementary Figure 1 Screening of toxic *Phytophthora* CRN effectors to *E. coli*.

Supplementary Figure 2 Screening of toxic *P. capsici* RxLR effectors to *E. coli*.

Supplementary Data Sheet 1 Strains and plasmids used in this study.

Supplementary Data Sheet 2 Primers used in this study.

## Abbreviations

CRN: crinkling- and necrosis-inducing protein
RxLR: Arg–Xaa–Leu–Arg
T4SS: type IV secretion system
T6SS: type VI secretion system
T4P: type IV pilus.
