# Supplementary material for: Filamentous Phytophthora Pathogens Deploy Effectors to Interfere With Bacterial Growth and Motility
Source: Front Microbiol. 2020 Sep 30;11:581511. doi: 10.3389/fmicb.2020.581511 (PMC7554372; doi:10.3389/fmicb.2020.581511)
Supplement: Supplementary Figure 1 — Screening of toxic Phytophthora CRN effectors to E. coli. [file Data_Sheet_1.doc]

**Supplementary materials**

**Filamentous *Phytophthora* pathogens deploy effectors to interfere with bacterial growth and motility**

Ji Wang, Danyu Shen, Chengcheng Ge, Yaxin Du, Long Lin, Jin Liu, Tian Bai, Maofeng Jing, Guoliang Qian*, Daolong Dou*


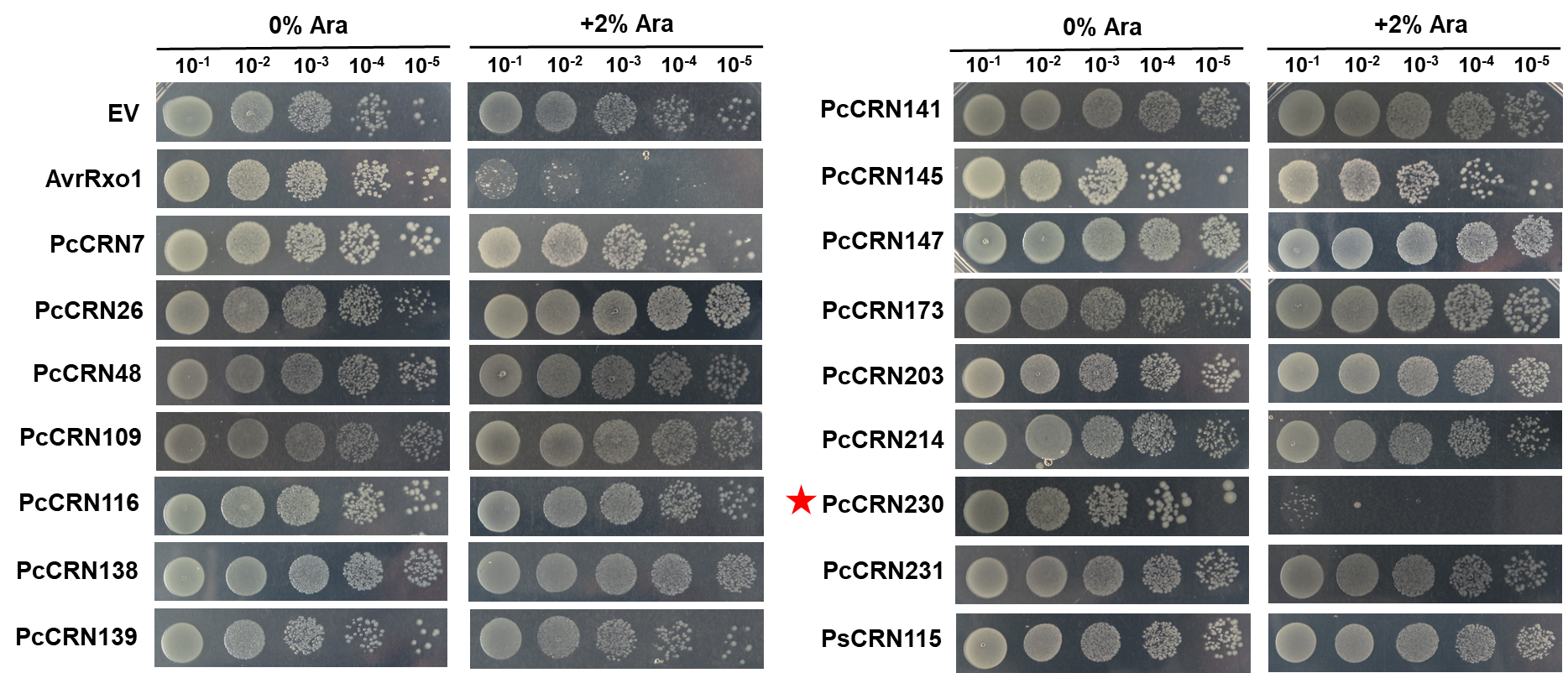


**Supplementary Figure 1 Screening of toxic *Phytophthora* CRN effectors to *E. coli*.** *E. coli* BL21(DE3) strains carrying designated pBAD-effector vectors were grown for several hours in LB broth at 37℃. Cultures were diluted to OD_600_=0.5, a 10 μL volume of each 10-fold serially diluted culture was spotted onto control plates or plates containing 2% arabinose. Plates were incubated at 37°C for 12 h and photographed. Empty vector was used as negative control, while AvrRxo1 was introduced as positive control. Results were repeated at three independent times.


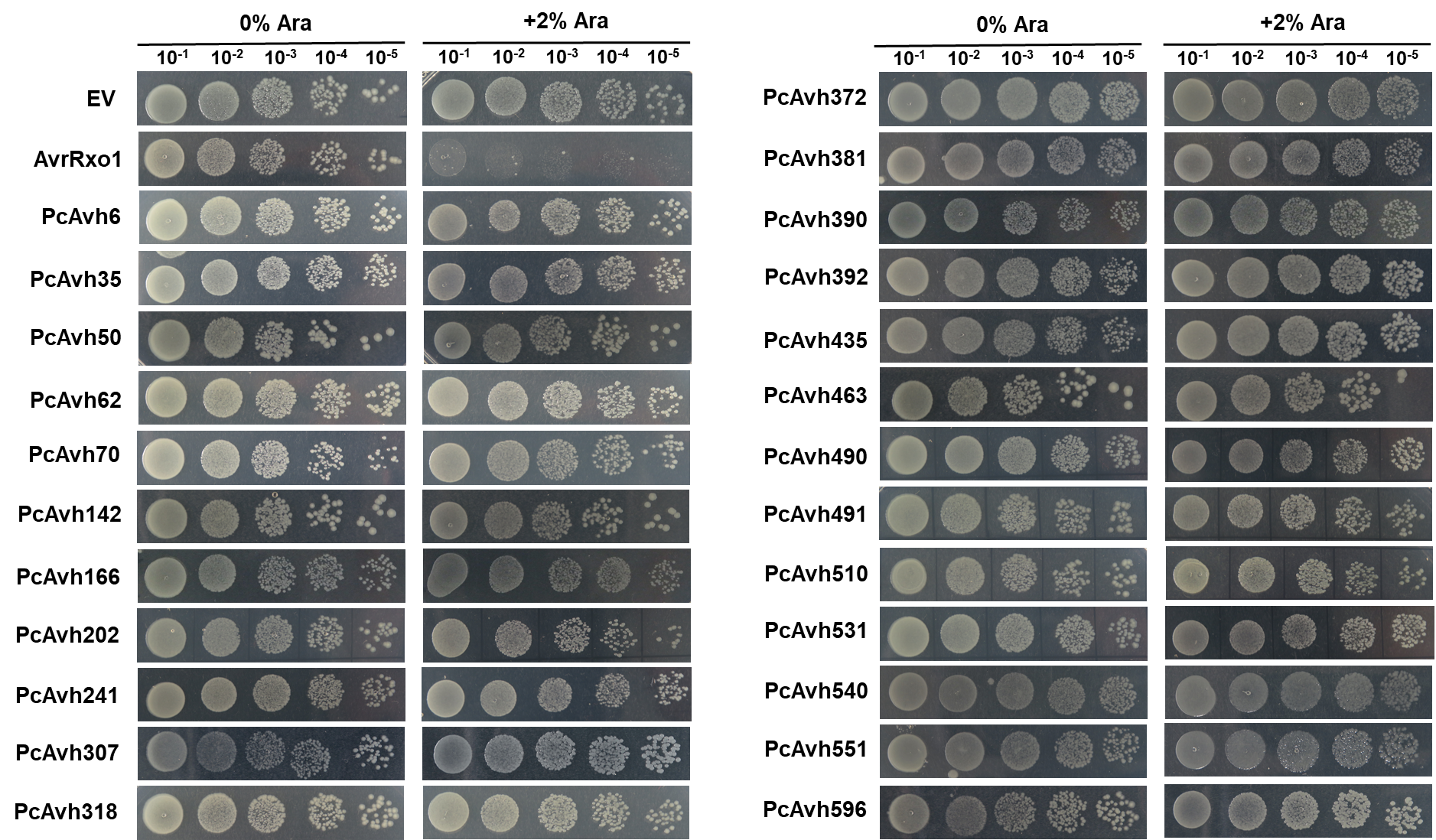


**Supplementary Figure 2 Screening of toxic *Phytophthora* *capsici* RxLR effectors to *E. coli*.** *E. coli* BL21(DE3) strains transformed with given effector pBAD vectors were cultured and serially diluted (10^-1^, 10^-2^, 10^-3^, 10^-4^ and 10^-5^), then spotted onto LB plates containing appropriate antibiotic and 2% arabinose. AvrRxo1 was used as positive controls, while empty vector as negative control. Plates were incubated at 37°C overnight and photographed. Three independent replicates were taken with same results.

**Supplementary Data Sheet 1. Strains and plasmids used in this study**

| **Strains and plasmids** | **Characteristics^a^** | **Source** |
| --- | --- | --- |
| ***Lysobacter enzymogenes*** | | |
| OH11 | Wild-type, Km^R^ | Qian et al. 2009 |
| ***Pseudomonas fluorescence*** | | |
| 2P24-LacZ | LacZ-labeled strain, Km^R^ | A gift strain from Professor Liqun Zhang and Xiaogang Wu |
| ***Escherichia coli*** | | |
| DH5α (pMD19-T) | DH5α harboring pMD19-T vector, LacZ-labeled strain, Am^R^ | TaKaRa Company, Dalian |
| MG1655 | Wild-type, host strain for protein expression in swimming motility assay | ATCC 700926a |
| *Trans1*-T1 Phage Resistant | Host strain for gene cloning, Am^R^ | TransGen Company, Beijing |
| BL21(DE3) | Host strain for protein expression, Km^R^ | TaKaRa Company, Shanghai |
| DH5α (pSMC21-GFP) | DH5α harboring pSMC21-GFP vector, Km^R^ | Lab collection |
| ***Agrobacterium tumefaciens*** | | |
| GV3101 | Host strain for gene transient expression in *N. benthamiana*, Km^R^, Rif^R^ | Lab collection |
| ***Phytophthora* pathogens** | | |
| *Phytophthora sojae* | P6497 | Lab collection |
| *Phytophthora capasici* | LT263 | Lab collection |
| *Phytophthora nicotianae* | Pp025 | Lab collection |
| **Fungal pathogen** | | |
| *Botrytis cinerea* | Wild-type | Lab collection |
| **Plasmids** | | |
| pBAD/Myc-His A | Vector for arabinose-inducible gene expression, Amp^R^ | Invitrogen |
| pSuper-RFP | Vector for RFP-labeled gene transient expression in *N. benthamiana*, Km^R^ | Lab collection |
| pBinGFP2 | Vector for GFP-labeled gene transient expression in *N. benthamiana*, Km^R^ | Lab collection |
| pBBR1-MCS5 | Broad-host-range vector for gene expression, Gm^R^ | Kovach et al. 1995 |

**Supplementary Data Sheet 2. Primers used in this study**

| **Primer** | **Sequence^a^** | **Purpose** |
| --- | --- | --- |
| **Primers used for gene induced expression in *E. coli*** | | |
| pBAD-AvrRxo1-F | GGggtaccATGAAAAACAAGACAGACATTGC | To amplify the coding region of *AvrRxo1* |
| pBAD-AvrRxo1-R | CCCaagcttGGAATTAGCTCGCTGTGAGCA |
| pBAD-PsCRN63-F | AGCTCGAGATCTGCAGCTggtaccATGTTTCCTGTGGACATTGACGC | To amplify the coding region of *PsCRN63* or *PsCRN115* without signal peptide |
| pBAD-PsCRN63/115-R | TTTTTGTTCGGGCCCaagcttGCTGAAATAACTAAACGTGGGAATAG |
| pBAD-PsCRN115-F | AGCTCGAGATCTGCAGCTggtaccATGTTCCCAGTGGACATCGATG |
| **Primers used for gene transient expression in *N. benthamiana* for protein extraction** | | |
| pBinGFP2-PsCRN63-F | GACGAGCTGTACAAGggtaccATGTTTCCTGTGGACATTGACGC | To amplify the coding region of *PsCRN63* or *PsCRN115* without signal peptide |
| pBinGFP2-PsCRN63/115-R | TCTAGTTCATCTAGAggatccGCTGAAATAACTAAACGTGGGA |
| pBinGFP2-PsCRN115-F | GACGAGCTGTACAAGggtaccATGTTCCCAGTGGACATCGATG |
| **Primers used for gene transient expression in *N. benthamiana* with RFP fluorescence** | | |
| pSuperRFP-PsCRN63QC-F | CAAATCGACTCTAGAaagcttATGGTGAAGCTCTTCTGTGCGATC | To amplify the full-length coding region of *PsCRN63* or *PsCRN115* |
| pSuperRFP-PsCRN63/PsCRN115-R | CTCGGAGGAGGCCATgtcgacGCTGAAATAACTAAACGTGGGA |
| pSuperRFP-PsCRN115QC-F | CAAATCGACTCTAGAaagcttATGGTGAAGCTATTCTGTGCGATA |
| **Primers used for PsCRN63 homologous gene induced expression in *E. coli*** | | |
| pBAD-PcCRN4-F | AGCTCGAGATCTGCAGCTggtaccATGGTGAAGCTTTCACTACAG | To amplify the coding region of *PcCRN4* |
| pBAD-PcCRN4-R | TTTTTGTTCGGGCCCaagcttACTGAAATAACTCAACGTGGGA |
| pBAD-PnSCP47-F | AGCTCGAGATCTGCAGCTggtaccATGCGAAGCGGGGTTATTC | To amplify the coding region of *PnSCP47* |
| pBAD-PnSCP47-R | TTTTTGTTCGGGCCCaagcttGCTGAAGTAACTCAGCGTGGG |
| **Primers used for *P. capsici* effector induced expression in *E. coli*** | | |
| pBAD-PcCRN7-F | TCGAGATCTGCAGCTggtaccATGGACAAAGAGACCGTCCAGC | To amplify the coding region of *PcCRN7* |
| pBAD-PcCRN7-R | TTTTTGTTCGGGCCCaagcttTGCCAAGGAGCTGCACATG |
| pBAD-PcCRN26-F | AGCTCGAGATCTGCAGCTggtaccATGATGAACGTGGAAATCGACCCG | To amplify the coding region of *PcCRN26* |
| pBAD-PcCRN26-R | TTTTTGTTCGGGCCCaagcttTGCGTAAGCGATCAACTTGACGATC |
| pBAD-PcCRN48-F | AGCTCGAGATCTGCAGCTggtaccATGAATGGATCGGGCGTCC | To amplify the coding region of *PcCRN48* |
| pBAD-PcCRN48-R | TTTTTGTTCGGGCCCaagcttCCGCGAAAGACACAGATTTGATC |
| pBAD-PcCRN116-F | AGCTCGAGATCTGCAGCTggtaccATGAGCCATGATTCCGTTCGACTGC | To amplify the coding region of *PcCRN116* |
| pBAD-PcCRN116-R | TTTTTGTTCGGGCCCaagcttCTTACGAACACGGGTCGGTGATG |
| pBAD-PcCRN138-F | AGCTCGAGATCTGCAGCTggtaccATGGAAGTGGTGAAGCTTTTCTG | To amplify the coding region of *PcCRN138* |
| pBAD-PcCRN138-R | TTTTTGTTCGGGCCCaagcttTGGCTGCGGATGTGAATCA |
| pBAD-PcCRN139-F | AGCTCGAGATCTGCAGCTggtaccATGGAGATCAATTCGAGGACCA | To amplify the coding region of *PcCRN139* |
| pBAD-PcCRN139-R | TTTTTGTTCGGGCCCaagcttTATCTCTTCAGGATACGGAACAAGAA |
| pBAD-PcCRN141-F | TCGAGATCTGCAGCTggtaccATGAATATCGTGCAGCTCAAAGAA | To amplify the coding region of *PcCRN141* |
| pBAD-PcCRN141-R | TTTTTGTTCGGGCCCaagcttCTGCGTCACGGGGGCAGC |
| pBAD-PcCRN145-F | AGCTCGAGATCTGCAGCTggtaccATGGTGACAGTCTTTTGTGCAATC | To amplify the coding region of *PcCRN145* |
| pBAD-PcCRN145-R | TTTTTGTTCGGGCCCaagcttCCTCCCCATCTCCATCCTATACA |
| pBAD-PcCRN147-F | AGCTCGAGATCTGCAGCTggtaccATGCGAACTGGAGCGATTCC | To amplify the coding region of *PcCRN147* |
| pBAD-PcCRN147-R | TTTTTGTTCGGGCCCaagcttAGTGCTATTCGCGAGCAAACC |
| pBAD-PcCRN173-F | AGCTCGAGATCTGCAGCTggtaccATGCCGATGCCAATGCTCTTCGGT | To amplify the coding region of *PcCRN173* |
| pBAD-PcCRN173-R | TTTTTGTTCGGGCCCaagcttTACTAATAGTTCGAGCAGCTCGT |
| pBAD-PcCRN203-F | AGCTCGAGATCTGCAGCTggtaccATGACAGTCATACATGACCGATACCG | To amplify the coding region of *PcCRN203* |
| pBAD-PcCRN203-R | TTTTTGTTCGGGCCCaagcttAACCCAGACACTCGTTGAAAGCATT |
| pBAD-PcCRN230-F | AGCTCGAGATCTGCAGCTggtaccATGCGTAAGGGTGAGAAGAGCTTC | To amplify the coding region of *PcCRN230* |
| pBAD-PcCRN230-R | TTTTTGTTCGGGCCCaagcttCTCATCCTGGTTGGAAGTAGCTG |
| pBAD-PcCRN231-F | AGCTCGAGATCTGCAGCTggtaccATGTGCCTTCATTTCCGCTTA | To amplify the coding region of *PcCRN231* |
| pBAD-PcCRN231-R | TTTTTGTTCGGGCCCaagcttATCCTCACCTGGTCGAAAATTCT |
| pBAD-PcAvh6-F | TCGAGATCTGCAGCTggtaccATGGCCCAAGTGCCACCA | To amplify the coding region of *PcAvh6* |
| pBAD-PcAvh6-R | TTTTTGTTCGGGCCCaagcttGAGACGACGTTCACGACGATTG |
| pBAD-PcAvh35-F | AGCTCGAGATCTGCAGCTggtaccATGGGACTGCAAGATCAAGCTGCG | To amplify the coding region of *PcAvh35* |
| pBAD-PcAvh35-R | TTTTTGTTCGGGCCCaagcttCAGGGGTTCCTTGTCGGGCA |
| pBAD-PcAvh50-F | TCGAGATCTGCAGCTggtaccATGGACAGTGATTCGAAGAGTATCTC | To amplify the coding region of *PcAvh50* |
| pBAD-PcAvh50-R | TTTTTGTTCGGGCCCaagcttTTGATTCTTAGTCCAAGTTGGATACTTT |
| pBAD-PcAvh62-F | AGCTCGAGATCTGCAGCTggtaccATGGTTAGTGATTCGAAGGGCC | To amplify the coding region of *PcAvh62* |
| pBAD-PcAvh62-R | TTTTTGTTCGGGCCCaagcttAACCGATGGGTTGCTGGTCCACG |
| pBAD-PcAvh142-F | TCGAGATCTGCAGCTggtaccATGGCGAATTCAGGTGGGA | To amplify the coding region of *PcAvh142* |
| pBAD-PcAvh142-R | TTTTTGTTCGGGCCCaagcttACCCAGCATGATCCTGATGTCG |
| pBAD-PcAvh202-F | AGCTCGAGATCTGCAGCTggtaccATGCTTTCGGCTCCCGTTGGCT | To amplify the coding region of *PcAvh202* |
| pBAD-PcAvh202-R | TTTTTGTTCGGGCCCaagcttCGCTTTTGTGGTCATCTTAGCGTAGT |
| pBAD-PcAvh241-F | AGCTCGAGATCTGCAGCTggtaccATGTACAGCGGTGCAGTCGAGTCT | To amplify the coding region of *PcAvh241* |
| pBAD-PcAvh241-R | TTTTTGTTCGGGCCCaagcttCCGCCCGTTGTCGCCCGTAC |
| pBAD-PcAvh307-F | TCGAGATCTGCAGCTggtaccATGCAAGTGGTCGCGCAA | To amplify the coding region of *PcAvh307* |
| pBAD-PcAvh307-R | TTTTTGTTCGGGCCCaagcttACCCATGGTCTTATACGTATCGTAGT |
| pBAD-PcAvh318-F | TCGAGATCTGCAGCTggtaccATGCATCCCGTGTATGAAGACTC | To amplify the coding region of *PcAvh318* |
| pBAD-PcAvh318-R | TTTTTGTTCGGGCCCaagcttACTGGTAGGAACAGCATTTTGCT |
| pBAD-PcAvh372-F | AGCTCGAGATCTGCAGCTggtaccATGTCATGTTCTCTTTCAGCGATGA | To amplify the coding region of *PcAvh372* |
| pBAD-PcAvh372-R | TTTTTGTTCGGGCCCaagcttAGAATAGTTTGCCGGTTGAGGTGATGA |
| pBAD-PcAvh381-F | AGCTCGAGATCTGCAGCTggtaccATGACAGTCTTGCCGGAGACG | To amplify the coding region of *PcAvh381* |
| pBAD-PcAvh381-R | TTTTTGTTCGGGCCCaagcttCCCTATCAAATCTCCAAGAGGACT |
| pBAD-PcAvh390-F | TCGAGATCTGCAGCTggtaccATGTTACCACGCCTTCGACTG | To amplify the coding region of *PcAvh390* |
| pBAD-PcAvh390-R | TTTTTGTTCGGGCCCaagcttACTCCATCTTGGTTCCAAAGTAAGC |
| pBAD-PcAvh435-F | AGCTCGAGATCTGCAGCTggtaccATGCGCTCTCGATGTCGTCGTC | To amplify the coding region of *PcAvh435* |
| pBAD-PcAvh435-R | TTTTTGTTCGGGCCCaagcttGTTCCTAGAGCGGTAATAAAGTAGGG |
| pBAD-PcAvh463-F | TCGAGATCTGCAGCTggtaccATGGAACTGCCCACCCGA | To amplify the coding region of *PcAvh463* |
| pBAD-PcAvh463-R | TTTTTGTTCGGGCCCaagcttAAGGAGGAGTGCCGTTTTGC |
| pBAD-PcAvh490-F | AGCTCGAGATCTGCAGCTggtaccATGGCTAACGCCCCGACAACCAG | To amplify the coding region of *PcAvh490* |
| pBAD-PcAvh490-R | TTTTTGTTCGGGCCCaagcttCGCCGCAGCAGAGGAGGTGGA |
| pBAD-PcAvh491-F | AGCTCGAGATCTGCAGCTggtaccATGGGCGAAAGCTACGAAAGC | To amplify the coding region of *PcAvh491* |
| pBAD-PcAvh491-R | TTTTTGTTCGGGCCCaagcttATGTGGCGCTACCGTGCC |
| pBAD-PcAvh510-F | AGCTCGAGATCTGCAGCTggtaccATGGCTCCGTTCAGTTCCTCAAT | To amplify the coding region of *PcAvh510* |
| pBAD-PcAvh510-R | TTTTTGTTCGGGCCCaagcttGTTAATCTGATTCTCCATGTCATCAGC |
| pBAD-PcAvh531-F | AGCTCGAGATCTGCAGCTggtaccATGGATGAAGCTGTGCTGGGTCTTG | To amplify the coding region of *PcAvh531* |
| pBAD-PcAvh531-R | TTTTTGTTCGGGCCCaagcttTGCAGCACGAACACTTTGGGTGT |
| pBAD-PcAvh540-F | AGCTCGAGATCTGCAGCTggtaccATGCTACGTCCACGTCTGCGTCCA | To amplify the coding region of *PcAvh540* |
| pBAD-PcAvh540-R | TTTTTGTTCGGGCCCaagcttCAAAATGACCACAAGGACAGCCG |
| pBAD-PcAvh551-F | AGCTCGAGATCTGCAGCTggtaccATGCCTGTGCGCGACGTC | To amplify the coding region of *PcAvh551* |
| pBAD-PcAvh551-R | TTTTTGTTCGGGCCCaagcttAACGTCTGTGTTGATCGGTTCG |
| pBAD-PcAvh596-F | TCGAGATCTGCAGCTggtaccATGATTGCAGTGACATTTTCCAGA | To amplify the coding region of *PcAvh596* |
| pBAD-PcAvh596-R | TTTTTGTTCGGGCCCaagcttCCTGTCCATCGCCCAGGA |
| pBAD-Slr-F | TCGAGATCTGCAGCTggtaccATGGAAGCTAAATTACCGCA | To amplify the coding region of *Slr-1143* |
| pBAD-Slr-R | TCGAGATCTGCAGCTggtaccGGTCTGCCAGTTGAAAATTGC |
| **Primers used for twitching motility assay** | | |
| PBBR-PcCRN173-F | TCCcccgggGATGCCGATGCCAATGCTCTT | To amplify the coding region of *PcCRN173* |
| PBBR-PcCRN173-R | GCtctagaCTACTTATCGTCGTCATCCTTGTAATCTACTAATAGTTCGAGCAGCT |
| PBBR-PcAvh540-F | TCCcccgggGATGCTACGTCCACGTCTGCG | To amplify the coding region of *PcAvh540* |
| PBBR-PcAvh540-R | GCtctagaCTACTTATCGTCGTCATCCTTGTAATCCAAAATGACCACAAGGACAG |
